# Thiophanate-methyl and its major metabolite carbendazim weaken rhizobacteria-mediated defense responses in cucumbers against Fusarium wilt

**DOI:** 10.1007/s42994-024-00181-5

**Published:** 2024-10-16

**Authors:** Kai Cui, Xiaoming Xia, Youwei Wang, Yueli Zhang, Ying Zhang, Junli Cao, Jun Xu, Fengshou Dong, Xingang Liu, Xinglu Pan, Yongquan Zheng, Xiaohu Wu

**Affiliations:** 1 State Key Laboratory for Biology of Plant Diseases and Insect Pests, Key Laboratory of Control of Biological Hazard Factors (Plant Origin) for Agricultural Product Quality and Safety, Ministry of Agriculture, Institute of Plant Protection, Chinese Academy of Agricultural Sciences, Beijing, 100193 China; 2 Institute of Quality Standard and Testing Technology for Agro-Products, Shandong Academy of Agricultural Sciences, Jinan, 250100 China; 3 College of Plant Protection, Shandong Agricultural University, Tai’an, 271018 China; 4 Institute of Plant Protection, Shandong Academy of Agricultural Sciences, Jinan, 250100 China

**Keywords:** Thiophanate-methyl and carbendazim, Rhizosphere microbiome, Cucumber Fusarium wilt disease, *Bacillus*, Ecotoxicological risk

## Abstract

The effect of fungicides on the plant-rhizosphere microbiome is a subject of ongoing debate, but whether any alteration in the rhizosphere microbiome could affect plant health is an issue that has not been thoroughly investigated. To address this deficiency, we analyzed the rhizosphere microbiome of wilt disease—resistant and disease-susceptible cucumber cultivars to determine whether (and which) plant-associated microorganisms have a role in disease resistance. We further assessed whether the fungicides thiophanate-methyl and carbendazim affect the rhizosphere microbiome, which may contribute to the plant’s immune response. Based on results acquired with both radicle-inoculation and soil-inoculation methods, cultivars Longyuanxiuchun (LYXC) and Shuyan2 (SY2) were identified as being disease resistant, whereas Zhongnong6 (ZN6) and Zhongnong38 (ZN38) were susceptible. The microbiome structure differed substantially between the resistant and susceptible plants, with LYXC and SY2 each having a significantly greater Shannon index than Zhongnong38. These results revealed that the disease-resistant cucumber cultivars recruited more beneficial bacteria, i.e., *Bacillus*, in their rhizosphere soil; as such, *Bacillus* was identified as a keystone genus in the microbial co-occurrence network. Thus, the presence of *Bacillus* may help cucumbers defend against fungal pathogens within the rhizosphere. *Bacillus subtilis* strain LD15, which was isolated from LYXC rhizosphere soil, could suppress pathogen growth, in vitro, and reduce disease severity in pot assays. Moreover, evidence also confirmed the accumulation of LD1 in the rhizosphere soil of resistant cucumber cultivars. For LYXC, application of thiophanate-methyl or carbendazim altered the microbiome structure, decreased bacterial diversity, and reduced the abundance of *Bacillus* species. Finally, pot assays verified that fungicide application decreased the proportion of LD15 in rhizosphere soil. From a microbial perspective, thiophanate-methyl and carbendazim may weaken the rhizobacteria-mediated defense response of cucumbers against cucumber Fusarium wilt disease. Our findings reveal a role for the rhizosphere microbiome in protecting plants from pathogens and constitute a reference for assessing the ecotoxicological risk of pesticides to non-target soil microorganisms.

**Supplementary Information:**

The online version contains supplementary material available at 10.1007/s42994-024-00181-5.

## Introduction

The rhizosphere microbiome interacts symbiotically with plants and thus provides a first line of defense against soil-borne pathogens to maintain plant health (Mendes et al. 2018; Sun et al. 2024). The rhizosphere microbiome helps maintain the health of host plants in two ways: collectively via competition with potentially pathogenic microorganisms, and specifically via the anti-pathogen activities of certain bacterial species (Cha et al. 2016). Research has suggested that the soil-borne diseases can be controlled—at least in part—by maintaining the inherent richness of the rhizosphere microbiome, or through increasing the abundance of certain beneficial bacterial species (Chen et al. 2020a; Kwak et al. 2018; Tao et al. 2020).

Chemical pesticides are the most common and effective means of controlling soil-borne pathogens. Most pesticides enter the soil directly following application, and indeed certain pesticides are applied directly via root irrigation (Hvězdová et al. 2018). Pesticide use, however, is oftentimes accompanied by changes in the soil microbiome, such as the relative abundance and functions of the numerous bacterial species that comprise the microbiome (Huang et al. 2021; Zhang et al. 2021). Still, whether any alteration of the microbiome is associated with the susceptibility of plants to disease has been less studied. Thus, it is of great significance to clarify the relationships between pesticides, the soil microbiome, and the incidence of plant diseases.

Cucumber Fusarium wilt disease, which is caused by *Fusarium oxysporum* f. sp. *cucumerinum* (FOC), is a destructive soil-borne vascular wilt disease. FOC can infect roots via uptake from the soil, eventually leading to plant death (Han et al. 2019). Thiophanate-methyl and its main metabolite carbendazim are the most commonly used fungicides for cucumber production, via root irrigation, but the effectiveness of pathogen control decreases year after year. One important reason is that pathogens may evolve resistance to a pesticide that is used continuously over multiple planting seasons, thereby increasing the relative abundance and longevity of the pathogen in soil (Chen et al. 2020b). A number of recent studies have reported that steering the soil microbiome can alter a plant’s defense against invading pathogen(s) (Liu et al. 2020; Wang and Li 2019; Wen et al. 2020; Ye et al. 2020). For instance, Wen et al. (2020) reported that certain organic acids, such as citric acid, pyruvic acid, succinic acid, and fumaric acid, may help enrich for species that benefit the rhizosphere microbiome; for example, species of the bacterial family *Comamonadaceae* may enhance the resistance of cucumbers to certain pathogens. Therefore, for another reason, we hypothesize that pesticides may disrupt plant-defense systems that reply on the rhizosphere microbiome. For example, carbendazim can decrease the diversity and metabolic activities of various soil microbes (Fang et al. 2016; Ma et al. 2021; Wang et al. 2009, 2012). Moreover, Fang et al. (2016) reported that the application of carbendazim could substantially reduce the abundance of species of certain beneficial bacterial genera, such as *Pseudomonas*, *Bacillus*, and *Burkholderia*. Therefore, to guide the use of pesticides in the field and ensure plant health, it is necessary to determine the changes in the rhizosphere microbiome after pesticide treatment and further elucidate the relationship between microbiome changes and plant immunity.

Yao and Wu (2010) showed that microbial biomass, microbial activities, and microbial communities can differ greatly in the rhizosphere of FOC-resistant and FOC-susceptible cucumber cultivars; they inferred that those differences may negatively affect the resistance of cucumber plants to Fusarium wilt. Owing to limitations in certain research techniques (e.g., phospholipid fatty-acid profiling), however, previous studies did not rigorously investigate the species composition of the entire cucumber rhizosphere and thus did not clarify which microbial species are key to disease resistance. Furthermore, it remains unknown whether the two fungicides thiophanate-methyl and carbendazim affect rhizomicrobial-mediated defense responses of cucumbers to Fusarium wilt disease. Here, we identified two cucumber cultivars with contrasting phenotypes, namely FOC resistance or susceptibility; these two cultivars were grown in two typical soils in the absence or presence of thiophanate-methyl or carbendazim, and the effects of each fungicide on the rhizosphere microbiome were assessed by sequencing amplicons of the 16S rDNA genes. The study aimed to address: (1) whether FOC-resistant cultivars have advantages over FOC-susceptible cultivars in terms of rhizosphere microbiome structure; (2) and if so, which microbial species are responsible for disease resistance; (3) and finally how does thiophanate-methyl or carbendazim affect the rhizosphere-microbiome with regard to plant immunity-based defense. The findings from our study help clarify the relationship between fungicides, the rhizosphere microbiome, and cucumber Fusarium wilt and will contribute to the efficient suppression of cucumber wilt disease.

## Results

### Cultivars LYXC and SY2 are more resistant to FOC than cultivars ZN6 and ZN38

**Fig. 1:**
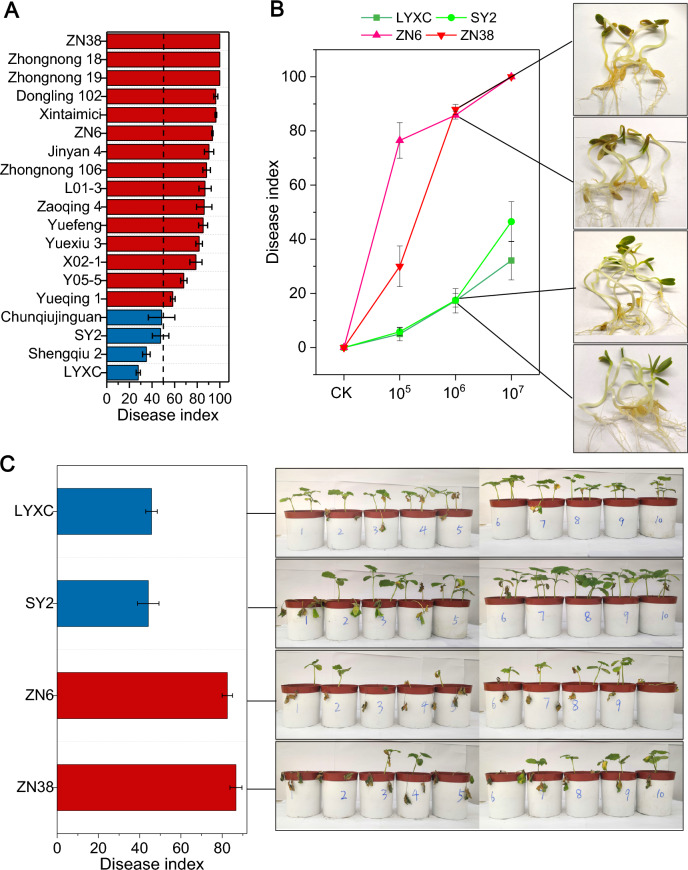
Resistance of different cucumber cultivars to Fusarium wilt disease. **A** The disease index for each of 19 cucumber cultivars after exposure to FOC via the radicle-inoculation method. Values are mean ± standard deviation (SD) from three replicates. **B**,** C** Disease index for each of four selected cucumber cultivars (LYXC, SY2, ZN6 and ZN38) that had been exposed to FOC via the radicle-inoculation method (**B**) or soil-inoculation method (**C**). Values are mean ± standard deviation (SD) from three replicates

The radicle-inoculation method was first used to evaluate the resistance of 19-different cucumber cultivars to FOC, and the disease indices ranged from 27.9 to 100 (Fig. 1A). Among all cultivars, LYXC and SY2 were identified as disease resistant, with disease indices of 27.9 and 47.6, respectively. Cultivars ZN6 and ZN38 were identified as disease susceptible, with a disease index of 93.4 and 100, respectively. The resistance of these four cultivars to FOC was further verified by the radicle-inoculation method at different inoculation concentrations of FOC (1 × 10^5^, 1 × 10^6^, and 1 × 10^7^ conidia/mL), and the results are shown in Fig. 1B. Again, LYXC and SY2 exhibited greater resistance than did ZN6 and ZN38 at each of the FOC concentrations; moreover, the disease index for each cultivar increased with increasing FOC concentration. The resistance of these four cultivars to FOC was further assessed by a soil-inoculation method. Similarly, in soil inoculated with FOC at 1 × 10^6^ conidia/g soil, LYXC and SY2 exhibited greater resistance than did ZN6 and ZN38 (Fig. 1C; disease index 45.7 for LYXC, 44.2 for SY2, 82.5 for ZN6, 86.7 for ZN38). At an FOC concentration of 1 × 10^4^ conidia/g soil, however, neither the FOC-resistant nor FOC-susceptible cultivars showed any obvious disease symptoms. Finally, these four cultivars were selected for subsequent experiments.

### The rhizosphere microbiome differs between FOC-resistant and FOC-susceptible cucumber cultivars

**Fig. 2:**
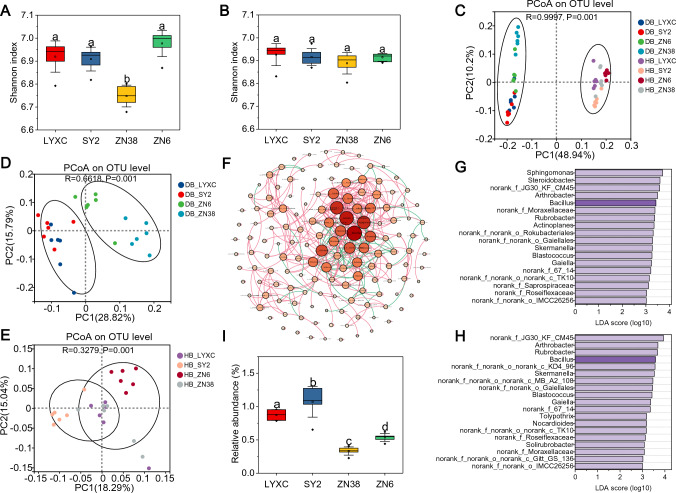
Comparison of the rhizosphere bacteria community between FOC-resistant and FOC-susceptible cucumber cultivars based on analysis of 16S rDNA amplicons (DB, black soil; HB, fluvo-aquic soil). **A**, **B** Bacterial diversity based on Shannon’s index in black soil (**A**) or fluvo-aquic soil (**B**). **C–E** Principal coordinates analysis (PCoA) applied to taxonomic composition at OTU levels for all soil samples (**C**) and two types of soils (**D**, **E**). **F** Co-occurrence networks of the rhizosphere bacteria at the genus level in black soil. **G**,** H** Linear discriminant analysis of effect (LEfSe) of the rhizosphere bacteria at the genus level in black soil between different cucumber cultivars: LYXC compared with ZN6 and ZN38 (**G**); SY2 compared with ZN6 and ZN38 (**H**). **I** Relative abundance of the *Bacillus* genus between different cucumber cultivars in black soil. In the box plots (**A**, **B** and** I**), the top and bottom of each box represent the 25th and 75th percentiles and the horizontal line inside each box represents the 50th percentile/median. The square point inside each box represents the average values and the error bars show the SD based on six replicates. The diamond point outside each box represents outliers. Different letters in each box plot indicate statistically significant differences (*P* < 0.05, one-way ANOVA, Duncan's test)

Microbial DNA sequences were obtained for 48 soil samples, and all statistical analyses were carried out based on rarefied OTU profiling at a depth of 36,904 sequences per sample. Results of the rarefaction curves (Fig. S1) indicated that the samples were adequate for subsequent analyses. According to alpha-diversity estimates, i.e., based on the Shannon index, the diversity of the bacterial community in the black-soil samples of the two FOC-resistant cultivars LYXC and SY2 was greater than that of the FOC-susceptible cultivar ZN38 (Fig. 2A); for the fluvo-aquic soil samples, however, the Shannon index did not differ significantly among the four cultivars (Fig. 2B). Principal coordinates analysis (PCoA) of the taxonomic composition at the OTU levels revealed that all soil-bacteria samples were first clustered according to soil type (Fig. 2C), then accordingly by a separation of cucumber cultivars (Fig. 2D, E). Moreover, the difference between the FOC-resistant and FOC-susceptible cultivars was more evident in the black soil, as confirmed by analysis of similarities. The rhizosphere bacteria may be specific to soils for cucumber cultivation. A co-occurrence network analysis was used to determine the complexity of connections within the rhizosphere microbiome for different cucumber cultivars in both the black and fluvo-aquic soil samples (Fig. 2F, Fig. S2). The degree of each genus was calculated using Gephi, and keystones were defined as the top 1% of degree in each co-occurrence network. As shown in Fig. 2F, *Bacillus* had the most degrees in the black soil, i.e., 27 (Table S1), which was important for maintaining the stability of the bacterial community and was the keystone in the black soil. More importantly, LEfSe and abundance analysis confirmed that the genus *Bacillus* was relatively more abundant for LYXC and SY2 in the black soil (Fig. 2G–I). Overall, these data suggested that the FOC-resistant cucumber cultivars may preferentially harbor a distinct set of rhizosphere bacterial species, such as *Bacillus*, which may affect plant resistance to FOC.

### *Bacillus* subtilis LD15 antagonizes FOC and suppresses cucumber wilt disease

**Fig. 3:**
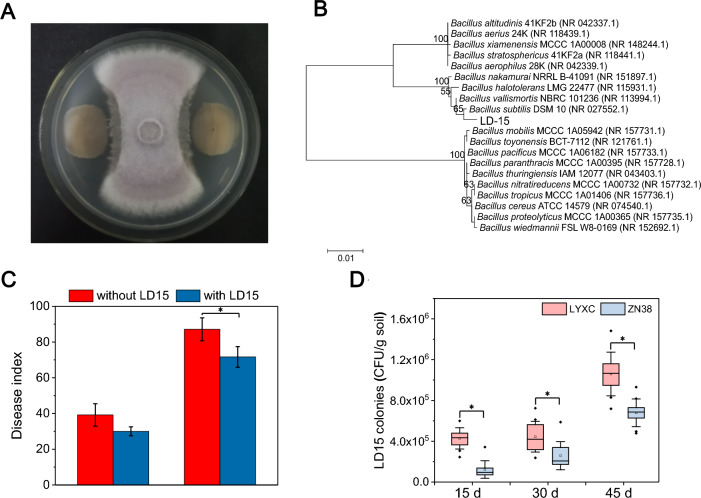
*Bacillus subtilis* LD15 and its effect on the progression of cucumber wilt disease. **A** Antagonism of FOC by strains of LD15 in culture plates. **B** Phylogenetic tree of strains of LD15 based on the sequence of the 16S rDNA. **C** Disease index for each of LYXC and ZN38 exposed to FOC in sterile soil treated with strains of LD15 or left untreated (control). *Indicates statistically significant differences based on three replicates (*P* < 0.05, Student’s *t* test). **D** Changes in the population of LD15^Rif^ in the rhizosphere soil of LYXC and ZN38 pretreated with LD15^Rif^. In the box plot (**D**), the top and bottom of each box represent the 25th and 75th percentiles and the horizontal line inside each box represents the 50th percentile/median. The square point inside each box represents the average values and the error bars show the SD based on ten seedlings. The diamond point outside each box represents outliers. *Indicates statistically significant differences based on ten seedlings (*P* < 0.05, one-way ANOVA, Duncan's test)

A total of 78 bacterial species were isolated from the black-soil rhizosphere, belonging to the genus *Bacillus* and to *Pseudomonas chlororaphis*, *Streptomyces netropsis*, and *Herbaspirillum huttiense* among species of other genera, of which LD15 had the most potent antifungal activity with a bacteriostatic rate of 67.5% in vitro (hyphal diameters: 29.3 ± 0.9 mm with LD15 and 90.0 ± 0.0 mm without LD15) (Fig. 3A). Based on sequence similarity of 16S rDNA, LD15 was identified as a *B. subtilis* sp. (Fig. 3B). The pot assay showed that LD15 had a clear capacity to reduce the severity of wilt disease, with disease indices of 30.0 for the disease-resistant cultivar LYXC and 71.7 for the sensitive cultivar ZN38, whereas the values for their respective control treatments were 39.2 and 87.1 (Fig. 3C), implying biocontrol efficacies of 17.7% and 23.4%. We further investigated the relative abundance of LD15 in the rhizosphere soil of different cucumber varieties. The mutant strain LD15^Rif^ was screened with a stable marker of the antibiotic rifampicin (100 mg/kg) using in a dual-culture experiment. In black soil, the LD15^Rif^ population in rhizosphere soil of LYXC was significantly larger than that of ZN38 (1.56−3.49 times) on days 15, 30, and 45 post-transplantation (Fig. 3D). These results indicated that disease-resistant cucumber cultivars may recruit a bacterial ally of LD15 to protect themselves from FOC infection.

### Thiophanate-methyl and carbendazim alter the bacterial composition and reduce the amounts of *Bacillus* in rhizosphere soil

**Fig. 4:**
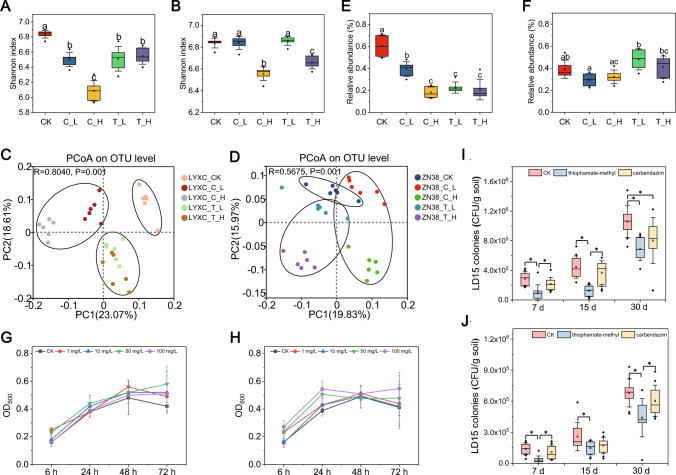
Effect of thiophanate-methyl and carbendazim on the rhizosphere bacteria community. **A**, **B** Bacterial diversity based on the Shannon index for LYXC (**A**) and ZN38 (**B**). **C**, **D** Principal coordinates analysis (PCoA) applied to taxonomic composition at OTU levels for LYXC (**C**) and ZN38 (**D**). **E**, **F** Relative abundance of the *Bacillus* genus for LYXC (**E**) and ZN38 (**F**). **G**, **H** Changes in the population of LD15^Rif^ in LB medium after pretreatment with thiophanate-methyl (**G**) or carbendazim (**H**). **I**, **J** Changes in the population of LD15^Rif^ in the rhizosphere soil of LYXC (**I**) and ZN38 (**J**) upon pretreatment with thiophanate-methyl and carbendazim. In the box plot (**A**, **B**, **E**, **F**, **I** and **J**), the top and bottom of each box represent the 25th and 75th percentiles and the horizontal line inside each box represents the 50th percentile/median. The square point inside each box represents the average values and the error bars show the SD base on six soil samples (**A**, **B**, **E** and **F**) or ten seedlings (**I** and **J**). The diamond point outside each box represents outliers. *Or different letters indicate statistically significant differences (*P* < 0.05, one-way ANOVA, Duncan's test). CK, control; T_L, inoculated with thiophanate-methyl at a low dose; T_H, inoculated with thiophanate-methyl at a high dose; C_L, inoculated with carbendazim at a low dose; C_H, inoculated with carbendazim at a high dose

Treatment of cucumber plants with a high dose of thiophanate-methyl and carbendazim resulted in a significant decrease in the Shannon index value for each of cultivars LYXC and ZN38; moreover, low-dose treatment with thiophanate-methyl and carbendazim also significantly decreased the index value for LYXC (Fig. 4A, B). Results of a PCoA revealed that application of thiophanate-methyl and carbendazim significantly altered the composition of the bacterial community in the rhizosphere soil of each of LYXC and ZN38, but the extent of the alteration varied with the amount of pesticide applied as well as with cucumber cultivar (Fig. 4C, D). The distance between the control and high-dose treatment was significantly greater than that between the control and low-dose treatment, especially for cultivar ZN38. Upon treatment with thiophanate-methyl and carbendazim, the relative abundance of *Bacillus* was significantly decreased for LYXC at both the low- and high-dose treatments (Fig. 4E); however, neither pesticide had a significant effect on ZN38 (Fig. 4F). Further analysis of the effect of thiophanate-methyl and carbendazim on the growth potential of LD15^Rif^ in vitro and in vivo revealed that neither pesticide had any obvious influence on LD15^Rif^ in vitro, as indicated by optical density (Fig. 4G, H). Conversely, in the in vivo study, the LD15^Rif^ population of LYXC was significantly decreased by thiophanate-methyl after 7, 15, and 30 days and by carbendazim after 30 days (Fig. 4I). For ZN38, the LD15^Rif^ population was significantly decreased by thiophanate-methyl after 7, 15, and 30 days and by carbendazim after 7 days (Fig. 4J). These results indicated that treatment of cucumbers with thiophanate-methyl and carbendazim could alter the structure of the rhizosphere bacterial community and thereby weaken the defense of plants against Fusarium wilt disease.

## Discussion

Throughout evolution, plants and pathogens have engaged in a continuous arms race for which plants have developed a complex and dynamic immune system to defend against pathogen invasion (Marín et al. 2013; Xie and Duan 2023; He et al. 2023). This immune system comprises not only plant-encoded components but also various biotic factors in the local environment, one of which is the rhizosphere microbiota (Chialva et al. 2022; Teixeira et al. 2019). Soil type is the main driving force that shapes the rhizosphere microbiome (Mahmud et al. 2021). In our study, PCoA revealed that all soil samples clustered first according to soil type, followed by a separation of cucumber cultivars. Similar to our findings, many studies have demonstrated that soil type has a greater effect than the plant genotype with respect to shaping the rhizosphere microbiome (Liu et al. 2019; Pérez-Jaramillo et al. 2019; Simonin et al. 2020). Many soil factors can potentially influence the taxonomic makeup of the rhizosphere microbiome, including soil texture, pH, organic matter content, nitrogen content, and abundance and type of metal ions (Li et al. 2018; Qi et al. 2018; Sullivan and Gadd 2019). Therefore, our results may be specific to soils for cucumber cultivation.

Our experiments with black soil revealed that disease-resistant cucumber cultivars may provide a rhizosphere environment that nurtures the growth of plant-friendly bacteria that can help defend against pathogen infection. On the one hand, resistant cultivars may simply recruit relatively larger numbers of soil bacteria to the rhizosphere. For instance, LYXC and SY2 each had a larger Shannon index than did ZN38. Empirical evidence and theoretical predictions suggest that greater bacterial diversity can decrease the success of pathogen invasion if such diversity leads to more efficient utilization of resources (Mendes et al. 2018; Wei et al. 2015). On the other hand, resistant cultivars may recruit certain beneficial bacteria, i.e., *Bacillus*, to alleviate the stress caused by pathogens. In our work, *Bacillus* was found to be the keystone genus, with the most degree (27), and had maximum connectivity with neighboring bacteria. A keystone species serves as a driver of soil-microbiome structure and function, highlighting the crucial roles of such species in maintaining the organization, integrity, and function of the entire microbial community (Banerjee et al. 2018; Chen et al. 2019). *Bacillus* species are well-known biocontrol agents that protect plants against root pathogens by several means, including their ability to antagonize pathogen success by competing for essential nutrients, to produce antifungal compounds, and to induce the plant immune response (Li et al. 2019; Khan et al. 2017). We found that the disease-resistant cucumber cultivars LYXC and SY2 had a significantly larger relative abundance of *Bacillus* species in the rhizosphere soil compared with the sensitive cultivars ZN6 and ZN38. Moreover, *B. subtilis* strain LD15, which was isolated from the rhizosphere soil of LYXC, was able to suppress FOC proliferation in vitro (bacteriostatic rate, 67.5%) and in vivo (i.e., pot experiments; biocontrol efficacy, 17.7 − 23.4%). We also confirmed that the rhizosphere soil of the disease-resistant cucumber cultivar LYXC had a larger abundance of LD15 than did the susceptible cultivar ZN38. Similar to our results, Kwak et al. (2018) found that the disease-resistant tomato variety Hawaii 7996 could recruit a larger relative proportion of *Flavobacterium* strain TRM1 to suppress the development of disease induced by *Ralstonia solanacearum* than the susceptible variety Moneymaker. Berendsen et al. (2018) reported that, upon foliar pathogen infection, *Arabidopsis thaliana* could recruit species from three bacterial genera, namely *Microbacterium*, *Stenotrophomonas*, and *Xanthomonas*, to the rhizosphere. Our findings support the assertion that disease-resistant cucumber cultivars may have co-selected certain bacterial species in their rhizosphere with functional traits that protect plants against pathogen invasion.

Cultivation of disease-resistant varieties combined with chemical control is one of the most effective means of preventing cucumber Fusarium wilt disease. In recent years, many studies have demonstrated that application of a fungicide(s) such as myclobutanil, difenoconazole, or mancozeb could alter the composition of the plant-rhizosphere microbiome and affect the abundance of certain bacterial species (Huang et al. 2021; Ju et al. 2016; Zhang et al. 2021). However, these reports did not include any in-depth discussion of whether these changes to the microbial structure were associated with plant health. In our present study, thiophanate-methyl and its major metabolite carbendazim could significantly alter both the bacterial community composition and structure; however, the relative influence of the alterations varied according to the level of pesticide application and cucumber cultivar. Notably, we established that both thiophanate-methyl and carbendazim significantly decreased bacterial diversity and the relative abundance of species of the keystone genus *Bacillus* for LYXC at doses typically applied in the field. Our in vitro experiment showed no obvious influence of thiophanate-methyl or carbendazim on LD15 growth; however, the results of the in vivo study confirmed the inhibitory effect of each of these pesticides on strain LD15. In addition to directly affecting the structure of the rhizosphere microbiome, pesticides may first act on the plants, which secrete rhizosphere metabolites to regulate soil microbial diversity (Bi et al. 2021). Moreover, pesticides may stimulate the growth of certain soil microorganisms that can outcompete and thereby inhibit the growth of LD15. From a microbial perspective, these two pesticides may weaken the rhizobacteria-mediated defense response of cucumbers against Fusarium wilt disease, especially for cultivar LYXC. As such, when assessing the appropriateness of applying a pesticide(s), any consideration of efficacy should also account for potential alterations in soil microorganisms and soil function with regard to maintaining plant health.

Our study has certain limitations. First, in addition to *B. subtilis* LD15, species of other genera or other *Bacillus* species may also contribute substantively to combatting Fusarium wilt disease, and thus further screening and verification of such species is required. Second, the soil samples utilized in our study were collected only during the cucumber flowering stage, during which the rhizosphere microbiome is relative stable, and this may have influenced the richness of bacterial species identified in the rhizosphere soil (Cao et al. 2020; Wen et al. 2020). Finally, how pesticides and plants together inhibit the growth of LD15 is a subject that will require intensive study and discussion.

Changes of microbial composition have been widely recognized due to the application of fungicides, but the relationship between microbial changes and plant health has been less discussed. Here, we identified the rhizosphere microbiome of cucumbers with contrasting phenotypes that developed in response to FOC challenge. Among 19-cucumber cultivars, LYXC and SY2 exhibited a higher level of FOC resistance compared with cultivars ZN6 and ZN38, and these four cultivars were selected as the test varieties. Our results indicate that the different cucumber cultivars had distinct rhizosphere microbiomes, and each rhizosphere microbiome was specific to the soil type used for cucumber cultivation. *Bacillus* was observed as the keystone genus to maintain the stability of the overall bacterial community. In addition, *Bacillus* was more abundant in the rhizosphere soil of FOC-resistant cucumber cultivars. Strain *B. subtilis* LD15, which was isolated from the rhizosphere soil of LYXC, could antagonize FOC in vitro and suppress cucumber wilt in pot experiments. Moreover, LD15 was confirmed to accumulate in the rhizosphere soil of the resistant cucumber cultivars. When applied at levels typically used for field crops, each of thiophanate-methyl and carbendazim altered the bacterial composition and decreased the bacterial diversity of the rhizosphere soil. Also, these pesticides reduced the abundance of *Bacillus* for LYXC and inhibited the growth of strain LD15. Our results demonstrate that thiophanate-methyl and carbendazim can weaken the rhizobacteria-mediated defense response of cucumber plants against Fusarium wilt disease and help clarify the potential negative effects of pesticide application on the rhizosphere microbiome and overall health of cucumber plants. This information will be useful for helping determine the rational use of pesticides and exploiting bacteria that are beneficial for protecting crops against pathogen invasion.

## Materials and methods

### Resistance of different cucumber varieties to FOC

#### Preparation of FOC

Samples of FOC were stored at − 80 °C in 25% aqueous glycerol (cryoprotectant). The FOC conidia were obtained as described by the National Agricultural Standards of China (NY/T 1857.3–2010) (http://down.foodmate.net/standard/sort/5/30375.html). Briefly, FOC was incubated on potato dextrose agar plates (200 g potatoes, 20 g glucose, 20 g agar, 1 L water) for 5 − 7 days at 28 °C, and then five agar blocks of mycelia (diameter, 5 mm) were excised and placed into flasks containing potato dextrose broth medium (200 g potatoes, 20 g glucose, 1 L water). The flasks were shaken at 180 rpm at 28 °C. After 7 days, the liquid culture was filtered through two layers of sterile gauze, and each filtrate constituted the suspension of conidia. Each suspension was centrifuged at 5000 r/min for 10 min, after which the supernatant was discarded. The residue was then suspended in sterile water, and FOC spores were quantified using a hemocytometer under a microscope.

#### Treatment of seeds

A total of 19-cucumber cultivars were collected from across China (see Table S2). For surface sterilization, the seeds were immersed first in 70% ethanol for 30 s followed by 5% aqueous sodium hypochlorite for 10 min, with final repeated washing (five times) in sterile distilled water. Sterilized seeds were placed on moist filter paper in the dark at 28 °C for germination. When the radicle length was approximately 0.5 − 1 cm, the germinated seeds were utilized for experimentation.

#### Inoculation of radicles with FOC

The radicle-inoculation method was conducted to evaluate the resistance of the 19-different cucumber cultivars to FOC. Initially, the germinated seeds were immersed in a suspension of FOC conidia (1 × 10^6^ conidia/mL) for 30 min. The FOC-inoculated seeds were then transferred into 15-cm culture dishes containing two layers of moist filter paper. As a control, seeds were treated with sterile water. All seeds were incubated in the dark at 28 °C for 10 days. Each treatment was replicated three times of 15 seeds each. The severity of cucumber disease symptoms was classified as shown in Table S3, and the disease index was then calculated using the formula as described by NY/T 1857.3–2010, i.e., disease index = [∑(rating × number of plants rated)/(total number of plants × highest rating)] × 100. Resistance of selected cucumber cultivars to FOC was further verified at different inoculation concentrations of FOC (1 × 10^5^, 1 × 10^6^, and 1 × 10^7^ conidia/mL). Cucumber disease symptoms and the disease index were recorded.

#### Inoculation of soil with FOC

The soil-inoculation method was further conducted to validate the resistance of selected cucumber cultivars to FOC. Germinated seeds were incubated in a greenhouse at 25 °C for 5−7 days. At the two-leaf stage, cucumber seedlings were carefully pulled from soil, and roots were thoroughly washed with sterile water to remove residual soil. The seedlings were then transplanted into pots containing soil inoculated with FOC, which had been prepared as follows. Nutrient-supplemented soil was first sterilized at 121 °C for 1 h. The suspended conidia were then added into the soil with thorough agitation. The final concentration of conidia was approximately 1 × 10^4^ or 1 × 10^6^ conidia/g soil. Each pot contained 150 g soil having a water content of ~ 60% by weight. After transplantation, the pots were kept in a greenhouse at 25 °C for 12 days, during which the plants were watered every 2 days (or as needed). Each treatment was replicated three times of 10 seedlings each. Cucumber disease symptoms were classified as shown in Table S3, and the disease index were calculated as described in section “Inoculation of radicles with FOC”.

### Analysis of the bacterial community between FOC-resistant and FOC-susceptible cucumber cultivars

#### Preparation of soil

To study the effect of soil on the rhizosphere microbiome, two typical soil types, namely black soil and fluvo-aquic soil, were respectively collected from Changchun city (N44°23′5″, E125°10′2″) and Langfang city (N39°30′55", E116°36′52"), China. The soil samples were taken from the 5- to 20-cm plough layer in fields that had no history of cucumber cultivation or fungicide application. The samples were homogenized and sieved through a 2-mm mesh to remove roots, stones, and other debris. The soil samples were incubated for 2 weeks to ensure recovery of the soil microbiome before the samples were used in pot experiments. Table S4 lists the physicochemical properties of the soils.

#### Pot experiment

A pot experiment was conducted to evaluate the rhizosphere bacterial communities of the four-selected cucumber cultivars with different resistance levels to FOC. All seedlings were grown in a greenhouse at the Institute of Plant Protection, Chinese Academy of Agricultural Sciences, Beijing, China (N40°03′22″, E116°29′27″). Each pot (height, 17.5 cm; diameter, 12.5 cm) was filled with ~ 2.0 kg soil into which four germinated seeds were sowed. Each cultivar was represented by 18 independent pots (six replicates, three pots for each replicate) per soil type, that is, four cultivars ×  two soil types ×  six replicates, resulting in 48 samples. The plants were grown at 25 − 30 °C with natural sunlight and watered every 2 days (or as needed). At the flowering stage (approximately day 45 after seeding, the plants were harvested and the roots shaken to remove loosely adhering soil. The rhizosphere soil was obtained by brushing off the soil attached firmly to the roots. The rhizosphere soil of 12 seedlings (3 pots) were combined into an individual sample (approximately 2.0 − 4.0 g in weight). Then, each sample was removed with impurities such as plant roots and larger clods for further use. Part of each soil sample was used for isolation of soil bacteria, and the remainder was stored at –80 °C for later extraction of DNA.

#### DNA extraction from soil samples and 16S rDNA amplicon sequencing

Total DNA was extracted from each soil sample (0.5 g) using the FastDNA® Spin kit for Soil (MP Biomedicals, USA). DNA quality and quantity were measured via electrophoresis through a 1% agarose gel and analysis with a NanoDrop™ 2000 spectrophotometer (Thermo Fisher Scientific, USA). The 16S rDNA partial gene was amplified using the primer set 338F/806R (338F-5'-ACTCCTACGGGAGGCAGCAG-3' and 806R-3'-GGACTACHVGGGTWTCTAAT-5') spanning ~ 468 bp of the V3–V4 region of the gene. PCR cycling was carried out as we previously reported (Rong et al. 2021), and amplified products were sequenced using the MiSeq PE300 sequencing platform (Illumina, USA) by Shanghai Majorbio Bio-pharm Technology (Shanghai, China).

#### Data processing

After sequencing the 16S rDNA amplicons, the raw data were quality-filtered using FASTQ (Chen et al. 2018) and assembled using FLASH (Magoč and Salzberg 2011) with the following three criteria. (1) The reads were truncated at any site receiving an average quality score of < 20 over a 50-bp sliding window. Further, reads were discarded if they contained ambiguous bases or were < 50 bp in length. (2) Only those sequences that overlapped by > 10 bp were assembled, with a mismatch ratio of ≥ 0.2. (3) Samples were identified based on barcodes and primers, and sequence directionality was subsequently deduced. The barcode was exactly matched, and primers were allowed a 2-nucleotide mismatch. All sequences were then clustered into operational taxonomic units (OTUs) at the 97% similarity level using UPARSE (Edgar 2013). Finally, the taxonomy of each OTU was matched using RDP Classifier (Wang et al. 2007) against the Silva v138 database with a confidence threshold value of 0.7. Prior to analysis, the 16S rDNA amplicon sequence for each sample was verified based a minimum number of sequence of all samples.

### Isolation and identification of rhizosphere *bacteria* and anti-FOC assays

#### Isolation and identification of rhizosphere *bacteria*

The soil samples used to isolate bacteria that could potentially antagonize FOC growth were the same as those used for the pot experiment (section “Pot experiment”). Total bacteria were extracted from soil as follows. Briefly, 10.0 g rhizosphere soil per sample was placed into a 250 mL flask, and 90 mL sterile water was added. Each flask was then shaken for 30 min at 180 rpm on a rotary shaker. After extraction, the suspension was serially diluted with sterile water, and 100 μL of each dilution was spread on an individual LB plate (LB: 5 g yeast extract, 10 g tryptone, 10 g NaCl, 20 g agar, 1 L water) in triplicate. All plates were incubated at 28 °C for 48 h. Morphologically distinct bacterial colonies, i.e., based on size, shape, margin, color, and growth pattern, were chosen for purification. The purified isolates were stored in 25% aqueous glycerol at – 20 °C.

#### Evaluation of the FOC-antagonistic activity of the bacterial isolates in vitro

The ability of each purified bacterial isolate to antagonize FOC growth in vitro was evaluated using a direct dual-culture method. Briefly, a single 7-mm-diameter cake of pathogenic fungus was placed in the center of a potato dextrose agar plate (diameter, 9 cm). Then, 0.5 μL of suspension of each bacterial isolate (10^7^ − 10^8^ colony-forming units [CFU] per mL) was spread approximately evenly at a distance of 2.5 cm around each fungal inoculum. All plates were incubated at 28 °C until the control-group plates (not treated with the isolates) were fully grown with fungus. The FOC-antagonistic activity was determined by measuring the diameter of the inhibition zone in each plate.

The antagonism-positive bacterial isolates were identified by 16S rDNA amplicon sequencing. Total DNA was extracted using a bacterial DNA isolation kit (Nanjing Vazyme Biotechnology Co. Ltd., Jiangsu, China). The 16S rDNA partial gene was amplified using the primer set 27F/1492R (5'-AGAGTTTGATCCTGGCTCAG-3' / 5'-GGTTACCTTGTTACGACTT-3') or (27F-5'-AGAGTTTGATCCTGGCTCAG-3' and 1492R-5'-GGTTACCTTGTTACGACTT-3') (Tan et al. 2019). PCR cycling was carried out as reported by Rameshkumar and Nair (2009). Amplified PCR products were sequenced by Beijing Ruiboxingke Biotechnology Co., Ltd. (Beijing, China). The bacterial species were then identified using the BLAST algorithm with reference to the GenBank database. A phylogenetic tree was constructed using the neighbor-joining method with Mega 7.0.

#### Evaluation of the FOC-antagonistic activity of the bacterial isolates in vivo

The FOC-antagonistic bacteria were incubated in LB medium at 28 °C for 48 h on a rotary shaker (180 rpm). Colonies were then harvested by centrifugation at 8000 r/min for 10 min and resuspended with sterile water. Each suspension was adjusted with sterile water to final concentration of 1 × 10^7^ CFU/mL. Cucumber seedlings of cultivars LYXC and Zhongnong38 (ZN38) at the two-leaf stage were soaked in each bacterial suspension for 1 min. The seedlings were then transplanted into soil that had been inoculated with FOC at concentrations of approximately 1 × 10^6^ conidia/g soil. The FOC-inoculated soil was prepared as described in section “Inoculation of soil with FOC”. Each treatment was replicated three times of ten seedlings each. After incubation for 12 days, disease symptoms for each cucumber group were recorded and the disease index was calculated.

#### Selection of a rifampicin-resistant mutant of LD15

The number of bacteria LD15 in the rhizosphere soil of each cucumber variety was determined by screening for rifampicin-resistant mutants of LD15 (termed LD15^Rif^) as described by Berendsen et al. (2018). Briefly, strain LD15 was transferred to LB medium containing increasing concentrations of rifampicin (1, 5, 10, 20, 40, 80 and 100 mg/L). LD15^Rif^ strains were then identified, and growth rates were found to be similar to that of wild-type LD15.

#### Recruitment of LD15^Rif^ by two cucumber varieties

A suspension of LD15^Rif^ bacteria was prepared as described in section “Evaluation of the FOC-antagonistic activity of the bacterial isolates in vivo”. The suspension was then mixed with with sterilize black soil (^60^Co γ-ray irradiation); the final density was 1 × 10^7^ CFU/g soil. Germinated seeds of cultivars LYXC and ZN38 were incubated in the LD15^Rif^-inoculated soil. On days 15, 30, and 45, the rhizosphere soil for each cucumber cultivar was collected, and LD15^Rif^ was isolated via water extraction from each soil sample as described in section “Isolation and identification of rhizosphere bacteria”. The suspension was serially diluted with sterile water, and 100 µL was spread on an individual LB plate containing rifampicin (100 mg/L). All plates were incubated at 28 °C for 48 h. Finally, the number of bacterial colonies on each plate was recorded.

### Effects of thiophanate-methyl and carbendazim on the cucumber-rhizosphere microbiome

#### Pot experiment

A pot experiment was conducted to evaluate the effect of thiophanate-methyl and carbendazim on the cucumber-rhizosphere microbiome. A single germinated seed for each of cultivars LYXC and ZN38 was sowed in sterile black soil (~ 250 g) in a single pot (10 cm high × 8 cm diameter). The pots were incubated at 25 °C with natural sunlight and watered every 2 days (or as needed). At the stage of two true leaves, each fungicide was applied via root irrigation. Plants were subjected to five distinct treatments, as follows: (1) not inoculated with any pesticide (control); (2) inoculation with thiophanate-methyl at 93.8 mg active ingredient (a.i.) per seedling (T-H); (3) inoculation with thiophanate-methyl at 9.38 mg a.i. per seedling (T-L); (4) inoculation with carbendazim at 93.8 mg a.i. per seedling (C-H); and (5) inoculation with carbendazim at 9.38 mg a.i. per seedling (C-L). The application dose of 93.8 mg a.i. of thiophanate-methyl for each seedling represents the recommended field-application rate for cucumbers. Before application, pesticides for each seedling were dissolved into 20 mL pure water. Each treatment comprised six replicates, and each replicate had eight seedlings. At the flowering stage, rhizosphere soil was collected for each treatment. DNA extraction and 16S rDNA amplicon sequencing and data processing were then conducted as described in sections “DNA extraction from soil samples and 16S rDNA amplicon sequencing” and “Data processing”.

#### Evaluation of the effect of pesticides on LD15^Rif^ in vitro

The effect of thiophanate-methyl and carbendazim on the growth of LD15^Rif^ in vitro was evaluated as described by Peng et al. (2014). Briefly, LD15^Rif^ was incubated in LB medium at 28 °C for 24 h on an orbital shaker (180 rpm). Then, 200 µL of each suspension was added into a new flask (50 mL) containing 20 mL LB medium with different concentrations of a fungicide (1, 10, 50, or 100 mg/kg). All flasks were incubated 28 °C on an orbital shaker (180 rpm). At 6, 24, 48 and 72 h, the optical density of each culture for each treatment was measured at 600 nm with an enzyme-labeled instrument.

#### Evaluation of the effect of pesticides on LD15^Rif^ in vivo

The effect of thiophanate-methyl and carbendazim on the growth of LD15^Rif^ was further evaluated in a pot experiment. Germinated seeds of cultivars LYXC and ZN38 were incubated in the LD15^Rif^-inoculated soil at a final density of 1 × 10^7^ CFU/g soil (the soil had been subjected to γ-irradiation with ^60^Co before use). At the stage of two true leaves, the pesticides were applied via root irrigation at 93.8 mg a.i. per seedling for each of the two pesticides. At days 15 and 30 after pesticide application, rhizosphere soil was collected for each treatment. Finally, the number of LD15^Rif^ colonies was determined via plate assay as described section “Inoculation of radicles with FOC”.

#### Data analysis

The α-diversity index, which reflects microbial community diversity in soil, was calculated using Mothur software. One-way analysis of variance with Duncan’s test was used to determine the statistical significance of differences between the alpha-diversity indices (Shannon index), relative abundance of *Bacillus* genus and population of LD15 colonies for different treatments using SPSS 22.0 software (IBM Corp., Armonk, NY, USA). Differences between taxonomic composition at OTU levels for different soil types, cucumber cultivars and pesticides were compared using principal coordinates analysis (PCoA) based on the Bray–Curtis distance. Linear discriminant analysis of effect size (LEfSe) was used to determine the number of distinct bacterial species among the various experimental groups, i.e., Biomarker, with a linear discriminant analysis threshold of > 3.0. To visualize interactions between different species, a co-occurrence network was constructed based on bacterial species with a relative abundance of ≥ 0.1% at the genus level. The correlation between genera was determined using the Spearman package in the R environment, and each network was visualized using Gephi (Spearman | r |> 0.8 and *P* < 0.01).

## Supplementary Information

Below is the link to the electronic supplementary material.

Supplementary file1 (DOC 475 KB)

## Data Availability

The data that support the findings of this study are available from the corresponding author upon reasonable request.
