# Supplementary material for: Thiophanate-methyl and its major metabolite carbendazim weaken rhizobacteria-mediated defense responses in cucumbers against Fusarium wilt
Source: aBIOTECH. 2024 Oct 16;5(4):417–30. doi: 10.1007/s42994-024-00181-5 (PMC11624165; doi:10.1007/s42994-024-00181-5)

**Thiophanate-methyl and its major metabolite carbendazim weaken rhizobacteria-mediated defense responses in cucumbers against Fusarium wilt**

Kai Cui^1, 2, #^, Xiaoming Xia^3,#^, Youwei Wang^3^, Yueli Zhang^4^, Ying Zhang^1^, Junli Cao^1^, Jun Xu^1^, Fengshou Dong^1^, Xingang Liu^1^, Xinglu Pan^1^, Yongquan Zheng^1^, Xiaohu Wu^1^, *

^1^ Institute of Plant Protection, Chinese Academy of Agricultural Sciences, State Key Laboratory for Biology of Plant Diseases and Insect Pests; Key Laboratory of Control of Biological Hazard Factors (Plant Origin) for Agricultural product Quality and Safety, Ministry of Agriculture, Beijing 100193, China

^2^ Institute of Quality Standard and Testing Technology for Agro-Products, Shandong Academy of Agricultural Sciences, Jinan 250100, China

^3^ College of Plant Protection, Shandong Agricultural University, Tai’an 271018, China

^4^ Institute of Plant Protection, Shandong Academy of Agricultural Sciences, Jinan 250100, China

^#^ These authors contributed equally to this work.

***Corresponding authors:**

Tel./fax: +86 10 62815938, E-Mail: xhwu@ippcaas.cn (X. Wu)

**Table captions**

**Table S1.** The top 15 bacterial genera with the most degrees in the two soils.

**Table S2.** Varieties and sources for different cucumber seeds.

**Table S3.** The disease degrees for different inoculation methods.

**Table S4.** Soil physichemical properties of two kinds of soils.

**Figure captions**

**Figure S1.** Rarefaction curves of the rhizosphere bacteria with different treatments.

**Figure S2.** Co-occurrence networks of the rhizosphere bacteria at the genus levels in the fluvo-aquic soil.

**Table S1.** The top 15 bacterial genera with the most degrees in the two soils.

| Rank | Black soil | | Fluvo-aquic soil | |
| --- | --- | --- | --- | --- |
|  | genus | degree | genus | degree |
| 1 | Bacillus | 27 | Leptolyngbya_FYG | 8 |
| 2 | norank_f__norank_o__Gaiellales | 25 | Leptolyngbya_ANT.L52.2 | 7 |
| 3 | norank_f__67.14 | 23 | Skermanella | 7 |
| 4 | Virgisporangium | 23 | Limnobacter | 7 |
| 5 | Cellvibrio | 20 | Blastococcus | 6 |
| 6 | norank_f__JG30.KF.CM45 | 19 | norank_f__JG30.KF.CM45 | 5 |
| 7 | Skermanella | 19 | Microvirga | 5 |
| 8 | Sporosarcina | 18 | Ramlibacter | 5 |
| 9 | Gaiella | 18 | Nodosilinea_PCC.7104 | 5 |
| 10 | norank_f__Geminicoccaceae | 17 | norank_f__Vicinamibacteraceae | 4 |
| 11 | Pseudomonas | 17 | norank_f__Geminicoccaceae | 4 |
| 12 | Candidatus_Alysiosphaera | 17 | unclassified_f__Nostocaceae | 4 |
| 13 | Rubrobacter | 16 | Cellvibrio | 4 |
| 14 | norank_f__norank_o__norank_c__TK10 | 16 | norank_f__norank_o__norank_c__bacteriap25 | 4 |
| 15 | Asticcacaulis | 16 | norank_f__norank_o__Azospirillales | 4 |

**Table S2.** Varieties and sources for different cucumber seeds.

| Number | Name | Source |
| --- | --- | --- |
| 1 | L01-3 | Horticultural Branch, Heilongjiang Academy of Agricultural Sciences, China |
| 2 | X02-1 | Horticultural Branch, Heilongjiang Academy of Agricultural Sciences, China |
| 3 | Y05-5 | Horticultural Branch, Heilongjiang Academy of Agricultural Sciences, China |
| **4** | **Longyuanxiuchun（LYXC）** | **Heilongjiang Longke Seed Industry Group Co. Ltd., China** |
| 5 | Shengqiu 2 | Heilongjiang Longke Seed Industry Group Co. Ltd., China |
| 6 | Chunqiujinguan | Shandong Xintai Xiangyun Seed Co., Ltd. China |
| 7 | Xintaimici | Shandong Xintai Xiangyun Seed Co., Ltd. China |
| 8 | Dongling 102 | Institute of Vegetables and Flowers of the Shandong Academy  of Agricultural Sciences, China |
| 9 | Jinyan 4 | Hebei Qingxian Aisen Vegetable Technology Promotion service Center, China |
| **10** | **Shuyan 2**  **（SY2）** | **Vegetable Research Institute, Hunan Academy of Agricultural Sciences, China** |
| 11 | Yuefeng | Vegetable Research Institute, Guangdong Academy of Agricultural Sciences |
| 12 | Yueqing 1 | Vegetable Research Institute, Guangdong Academy of Agricultural Sciences |
| 13 | Yuexiu 3 | Vegetable Research Institute, Guangdong Academy of Agricultural Sciences |
| 14 | Zaoqing 4 | Vegetable Research Institute, Guangdong Academy of Agricultural Sciences |
| **15** | **Zhongnong**  **（ZN6）** | **Institute of Vegetables and Flowers of the China Academy of Agricultural Sciences, China** |
| 16 | Zhongnong 18 | Institute of Vegetables and Flowers of the China Academy of Agricultural Sciences, China |
| 17 | Zhongnong 19 | Institute of Vegetables and Flowers of the China Academy of Agricultural Sciences, China |
| **18** | **Zhongnong 38**  **（ZN38）** | **Institute of Vegetables and Flowers of the China Academy of Agricultural Sciences, China** |
| 19 | Zhongnong 106 | Institute of Vegetables and Flowers of the China Academy of Agricultural Sciences, China |

**Table S3.** The disease degrees for different inoculation methods.

| Degree | Radicle-inoculated method | Soil-inoculated method |
| --- | --- | --- |
| 0 | Healthy | Healthy |
| 1 | Hypocotyl or cotyledons have slightly withered appearance, cotyledons lose their luster | Cotyledons turn yellow, but not wilted |
| 2 | Plants have slightly withered appearance, hypocotyl have necrotic spots or cotyledons turn yellow | Cotyledons turn wilted |
| 3 | Plants have moderately withered appearance, cotyledons get drooped | Cotyledons and true leaves turn wilted, or  plants are dwarfed |
| 4 | Plants have seriously withered appearance, plants fall and die | Plants fall and die |

**Table S4.** Soil physichemical properties of two kinds of soils

| Physichemical property | Fluvo-aquic soil | Black soil |
| --- | --- | --- |
| Organic matter (g/kg) | 14.26 | 17.63 |
| pH | 7.3 | 7.0 |
| Available phosphorus (mg/kg) | 3.51 | 7.63 |
| Available potassium (mg/kg) | 396.60 | 179.44 |
| Total nitrogen (%) | 0.11 | 0.15 |

**Figure S1.** Rarefaction curves of the rhizosphere bacteria with different treatments.


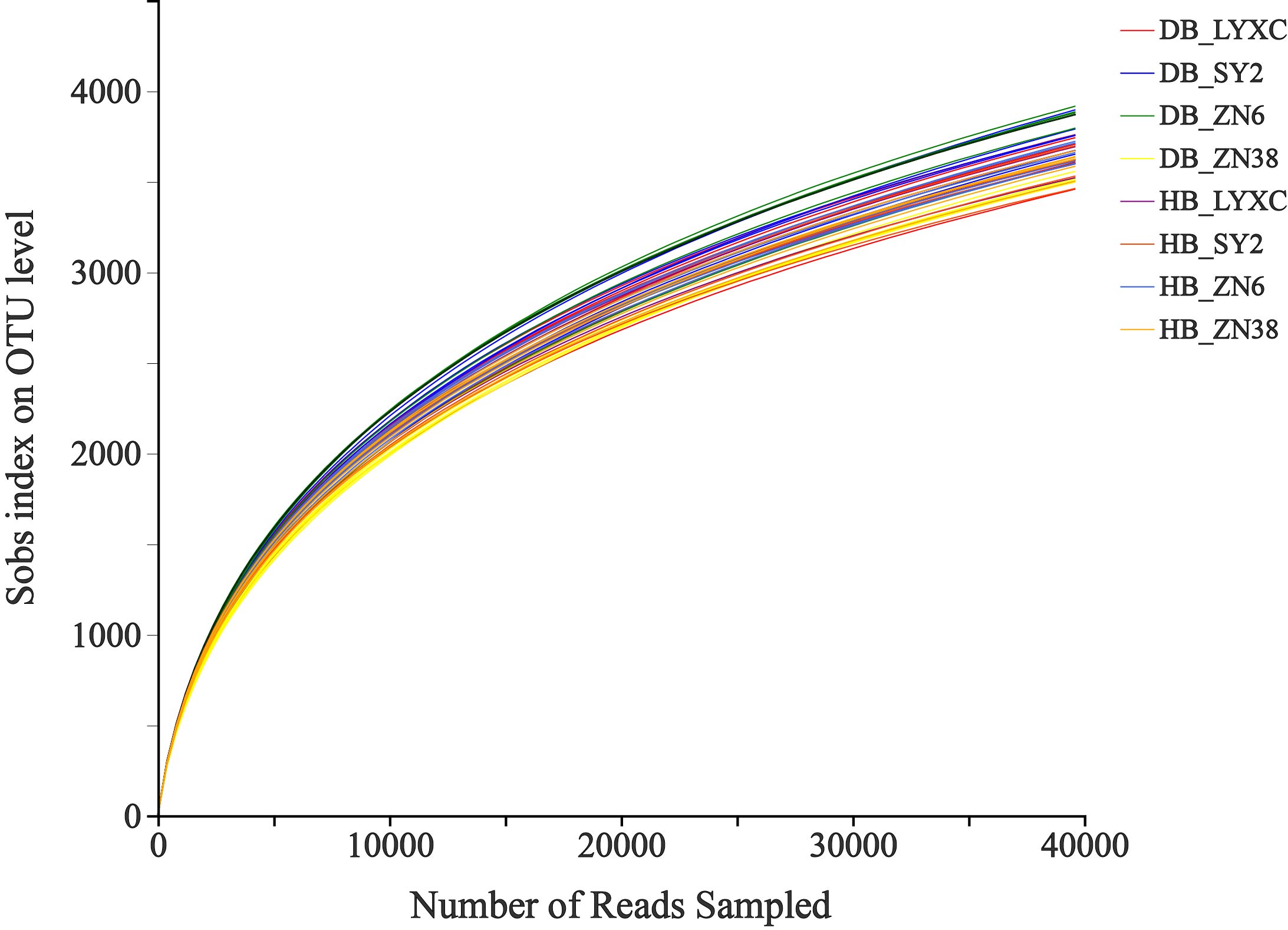


**Figure S2.** Co-occurrence networks of the rhizosphere bacteria at the genus levels in the fluvo-aquic soil.


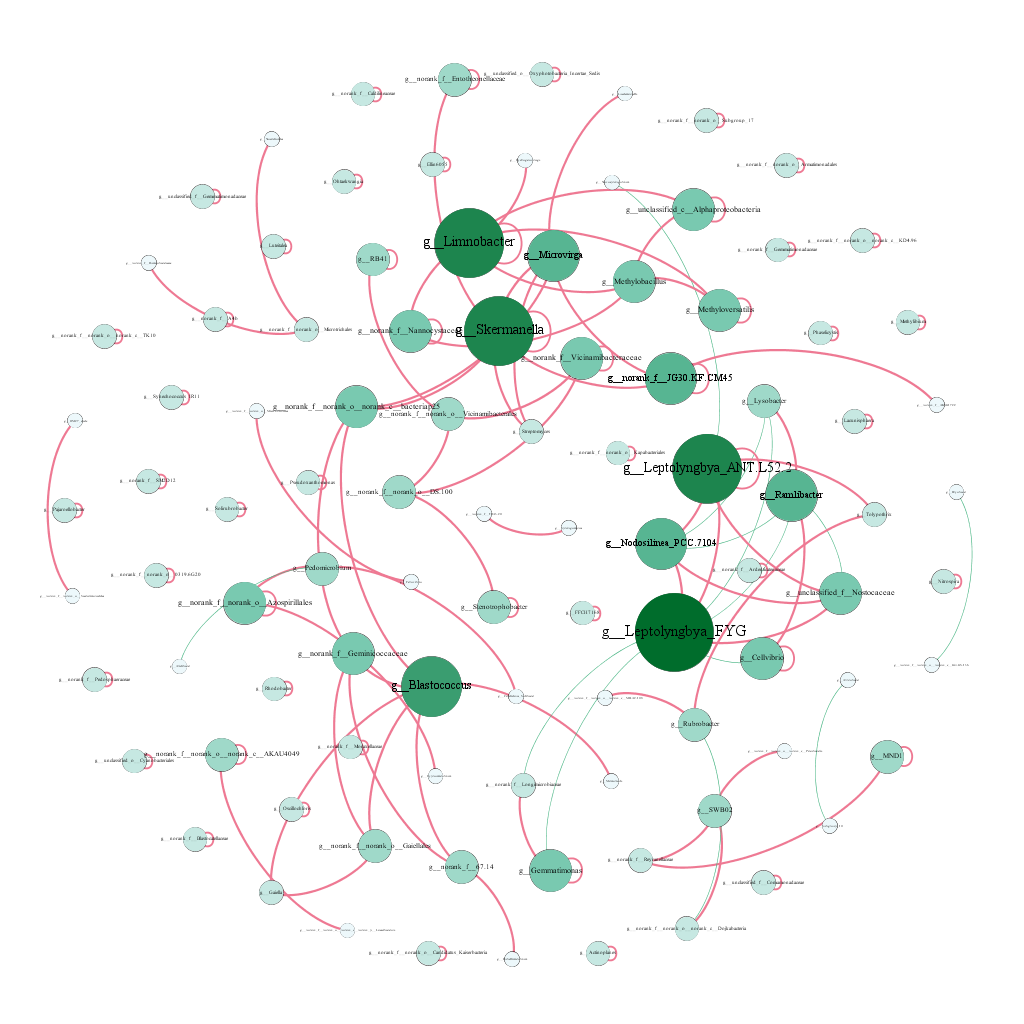

Supplement: Supplementary file 1 — Supplementary file1 (DOC 475 KB) [file 42994_2024_181_MOESM1_ESM.doc]
